# Chitosan-Based Nanoparticles Containing Cherry Extract from *Prunus avium* L. to Improve the Resistance of Endothelial Cells to Oxidative Stress

**DOI:** 10.3390/nu10111598

**Published:** 2018-11-01

**Authors:** Denise Beconcini, Angela Fabiano, Ylenia Zambito, Roberto Berni, Tatiana Santoni, Anna Maria Piras, Rossella Di Stefano

**Affiliations:** 1 Department of Surgery, Medical, Molecular, and Critical Area Pathology, University of Pisa, via Paradisa 2, 56100 Pisa, Italy; denisebeconcini@gmail.com (D.B.); t.santoni@ao-pisa.toscana.it (T.S.); rossella.distefano@unipi.it (R.D.S.); 2 Department of Pharmacy, University of Pisa, via Bonanno 33, 56100 Pisa, Italy; angyfab@gmail.com (A.F.); anna.piras@unipi.it (A.M.P.); 3 Department of Life Sciences, University of Siena, via P.A. Mattioli 4, 53100 Siena, Italy; berni10@student.unisi.it; 4 Interdepartmental Research Center Nutraceuticals and Food for Health, University of Pisa, via Borghetto 80, 56100 Pisa, Italy; 5 Trees and Timber Institute-National Research Council of Italy (CNR-IVALSA), via Aurelia 49, 58022 Follonica (GR), Italy

**Keywords:** chitosan derivatives, sweet cherry (*Prunus avium* L.), bioactive properties, cardiovascular diseases, polyphenols, antioxidant activity, HUVECs, oxidative stress

## Abstract

Cherries are known for their nutraceutical properties, in particular for their antioxidant ability due to their polyphenol content, which causes a reduction of cardiovascular disease (CVD) risk factors. However, once ingested these molecules are degraded in the Gastrointestinal (GI) tract before reaching the blood, which is the action site. The object of the present work is to evaluate the ability of cherry extract (CE), encapsulated in nanoparticles (NPs) based on different chitosan (Ch) derivatives, to promote a protective effect of human umbilical vein endothelial cells (HUVECs) involved in vascular dysfunction against oxidative stress. CE-loaded NPs based on quaternary ammonium chitosan (NP1) and an S-protected thiolated derivative thereof (NP2) were prepared. The mean particle size (NP1 344.9 ± 17.8, NP2 339.9 ± 68.2 nm), the polydispersity index, the encapsulation efficiency (NP1 78.4 ± 4.5, NP2 79.8 ± 0.6%), and the zeta potential (NP1 14.8 ± 0.3, NP2 15.8 ± 0.5 mV) did not appear to be significantly different. Both NP types improved the CE apparent permeation parameters with respect to the control. Conversely, CE-loaded NP2 protected HUVECs from oxidative stress and reduced reactive oxygen species (ROS) production more than CE-loaded NP1 and free CE. In addition to promoting HUVEC resistance, NP2 could be a useful tool to overcome the problem of cherry seasonality.

## 1. Introduction

Nutraceuticals and functional foods have raised considerable interest due to their presumed safety and potential nutritional and therapeutic effects [1]. The consumption of plants derivatives products has showed a very important role in reducing physiological threats thanks to the action mechanism of their chemical compounds, such as the improvement in immune responses and defense system [2,3,4].

Among plant products, cherry fruits have widely been described for their nutritional properties and beneficial effects of their compounds on human health [5]. In particular, the chemical composition and food applications of sweet cherries have recently been reported [6]. The most representative molecules in cherries are polyphenols such as flavonoids and anthocyanins, which represent the most abundant antioxidants in the diet [7]. Their antioxidant and anti-inflammatory properties have previously been described [8,9]. Cherry extracts (CEs) are characterized by a high content in phenols and consequently high nutraceutical potential, which could prevent chronic diseases.

Epidemiological studies have suggested that fruit consumption was related to a reduction in cardiovascular disease (CVD) risk factors [10]. It has also been shown that cherry consumption plays an important role in the struggle against inflammatory diseases, selectively reducing several biomarkers associated therewith [11]. Among CVDs, atherosclerosis is considered to be one of the most important causes of human mortality. Smoking, hypertension, diabetes, obesity, and above all oxidative stress demand a restorative response from the vascular wall by means of the inflammatory process, causing the outbreak and progression of atherosclerotic disease [12]. Endothelial cells that line the blood vessels are involved in the regulation of both vascular tone and blood vessel permeability, and are very sensitive to injury caused by oxidative stress [13]. At high concentrations, reactive oxygen species (ROS) can cause severe damage to cellular structures and components, leading to cell death [14].

Since non-commercial fruits typical of a regional territory contain nutraceuticals and are characterized by a higher phenolic content compared to commercial fruits [15], we chose ancient varieties of sweet cherries from *Prunus avium* L., thriving in Tuscany, which represent an interesting source of bioactives to study [16]. In particular, the Crognola tuscan cherry variety was used to evaluate the protective effect exerted by the extracted polyphenols on endothelial cells’ oxidative stress. However, a major drawback of using antioxidants extracted from fruits is in their low bioavailability. Indeed, they are characterized by poor intestinal absorption, high liability to oxidation in the intestinal tract and metabolic degradation in liver. Hence, effective concentrations of these substances have a poor probability of being found in the organism or in the blood, i.e., their site of action, for a long time after ingestion.

From here the idea descended of preparing cherry nanoparticles (NPs), which showed promise for prolonging the residence time of polyphenols in the Gastrointestinal (GI) tract, so decreasing the importance of the intestinal clearance mechanisms and increasing the surface available for the interaction with the biological target. Furthermore, the NPs are able to penetrate into tissues via the capillaries and to be internalized by cells. Our recent studies have shown that chitosan derivative-based NPs are able to promote the absorption of grape extracts [17,18] and, once internalized by enterocytes, are able to cross, unaltered, the intestinal epithelium and reach the blood, i.e., the target site. More mucoadhesive NPs showed a greater ability to enhance the bioavailability of the encapsulated drug than less mucoadhesive ones [19].

The object of the present work has been to evaluate the ability of Crognola cherry extract (CE), encapsulated in NPs based on different chitosan (Ch) derivatives, to promote a protective effect of cells, e.g., human umbilical vein endothelial cells (HUVECs) involved in vascular dysfunction [20,21,22], against oxidative stress.

## 2. Materials and Methods

### 2.1. Polymeric Materials

The polymer derivatives used in this study were prepared according to the methods described in the references cited: reduced-MW (Molecular weight) hyaluronic acid (rHA (Reduced-MW hyaluronic acid), viscosimetric MW 470 kDa) [23]; quaternary ammonium-Ch conjugates synthesized at 60 °C, coded as QA-Ch [24]; and a thiolated derivative of QA-Ch, coded as QA-Ch-S-pro [19].

### 2.2. Fruits Collection and Cherry Extract (CE) Preparation

Crognola Capannile cherry fruits (*Prunus avium* L.) were harvested at their highest degree of maturation (1 June–25 July 2016) from plants present in the Tuscan germplasm collection of the Santa Paolina experimental farm, which is part of National Research Council of Italy—Trees and Timber Institute (*CNR-IVALSA*) located in Follonica (Italy). The farm is a section of the Regional Genetic Bank. After collection, fruits were put in a refrigerator at −80 °C to stop their normal biological processes. In accordance with Abozed et al. [25], the extraction of the molecules of interest was carried out using frozen fruit. The fruit was crumbled and weighed (3 g) and 6 mL of acetone 70% *v/v* were added. Then the solution was homogenized (Ultra-Turrax^®^ T25 basic IKA, Saint Louis, MS, USA) for about 3 min and sonicated (Elma Transsonic T 460/H, Wezikon, Switzerland) for 20 min to ensure complete cellular breakdown. The solution was homogenized again for one minute and centrifugated for 5 min at 13,000 rpm (Eppendorf^®^ centrifuge 5415D, Hamburg, Germany) to separate the phenolic compounds from the pellet. The obtained supernatant was filtered with a 0.45-μm cellulose acetate membrane filter (Sartorius, Göttingen, Germany) and then dried (Jouan Model RC10-10 Concentrator Centrifuge, Milano, Italy) to remove acetone. At the end, the cherry extract (CE) was freeze-dried. The freeze-dried samples were stored at −20 °C.

### 2.3. Antioxidants Determination

The total antioxidant potential of CE freeze-dried samples was determined using the ferric-reducing antioxidant power (FRAP) assay reported by Benzie and Strain [26]. This is based on the reduction of Fe^3+^-2,4,6-Tri(2-pyridyl)-s-triazine (TPTZ) to a blue-colored Fe^2+^-TPTZ. The absorbance was read at 593 nm (Perkin Elmer, Lamba 25 spectrophotometer, Waltham, MA, USA). The FRAP value of the samples, expressed as mg of Fe^2+^ per mL, was determined from a standards curve built up using ferrous sulphate.

### 2.4. Total Polyphenolic Content

The total polyphenol content (TPC) of CE samples, before and after freeze-drying, was determined by the spectrophotometric method of Folin–Ciocalteau [27]. The results were expressed as gallic acid equivalent (GAE) on a dry weight basis. The TPC of CE was correlated with concentration by reacting standards of known CE concentration in phosphate buffer pH 7.4 (PB, 0.065 M), with the Folin–Ciocalteau reagent and measuring the resulting absorbance at 765 nm, as previously described [17,28]. A linear absorbance-concentration correlation was found (*r*^2^ = 0.998, *n* = 6) which allowed determination of unknown CE sample concentration.

### 2.5. Preparation and Characterization of CE-Loaded NPs Based on Chitosan Derivatives

To prepare CE-loaded NPs based on QA-Ch and QA-Ch-S-pro, a 650-μL volume of PB containing 0.05 mg/mL rHA and 4.5 mg/mL CE was added portion-wise (50 μL) to 5 mL of 0.5 mg/mL Ch derivative solution in PB under stirring. The resulting particles were analyzed for size by light scattering (Beckman/Coulter N4 plus, Brea, CA, USA) and ζ-potential (Litesizer™ Anton Paar, Graz, Austria). Blank NPs were prepared by the same procedure, except that the rHA solution contained no CE. Following preparation, each CE-loaded and blank NP dispersion was centrifuged (20,000 rpm, 1 h, 4 °C) and the supernatant analyzed by the Folin–Ciocalteau reagent for TPC, as described in Section 2.4. To obtain the CE amount in the supernatant (CE_sup_) the absorbance for the blank NPs was subtracted from that of CE-loaded NPs. The NP entrapment efficiency (EE) was calculated by the following equation:

EE = (CE_tot_ − CE_sup_)/CE_tot_. (1)

where CE_tot_ represents the total CE mass used for NP preparation.

### 2.6. CE Stability Studies

The stability of CE that was free or encapsulated in NPs at pH 1.2 was evaluated according to the following procedure. CE-loaded NPs prepared as described in Section 2.5. and a solution of CE in PB were acidified to pH 1.2 with HCl 1 M and immersed in a water bath at 37 °C under continuous stirring to simulate gastric fluids (SGF). At 30 min intervals of a total of 240 min, 50 μL volume was withdrawn and analyzed by the Folin–Ciocalteau reagent for TPC, as described in Section 2.4.

### 2.7. Ex Vivo Permeation Experiments

A previously described procedure was followed [28]. The intestinal mucosa used for these studies was excised from non-fasting male Wistar rats weighing 250–300 g. One and 3 mL of phosphate buffer pH 7.4, 0.13 M, isosmotic (PB, PH = 7.4) were added to the mucosal (donor) and serosal (acceptor) compartments, respectively, of the permeation cell. The Ussing chamber was then placed in a thermostatic water bath at 37 °C. After 20 min equilibration the medium in the donor compartment was replaced with 1 mL of each pre-equilibrated NP dispersion, or a CE solution (control) (15.5 µg/mL GAE concentration). The apical to basolateral transport of CE was investigated, i.e., at 30 min intervals, for a total of 240 min; 50 μL volume was withdrawn from the acceptor compartment and replaced with an equal volume of fresh pre-equilibrated PB (PH = 7.4). The withdrawn samples were analyzed for TPC by the Folin–Ciocalteau reagent. Three or more repeats were carried out for each experiment. In order to avoid any possible analytical interference, 3 blank runs were performed as described above and the mean value obtained at each time point was subtracted from that obtained at each corresponding time with the samples under study.

### 2.8. Human Umbilical Vein Endothelial Cell (HUVEC) Isolation and Culture

Human umbilical vein endothelial cells (HUVECs) are cells derived from the endothelium of veins from healthy human donor umbilical cords, following the procedure described by Jaffe et al. [29]. Isolated HUVECs were centrifuged and the cell pellet was plated on gelatin pre-coated flasks and incubated for 24 h at 37 °C, 5% CO_2_ in growth medium consisting of Medium 199 (M199) (PAN-Biotech, Dorset, UK), fetal bovine serum (FBS) (PAN-Biotech, Dorset, UK), penicillin–streptomycin solution (PAN-Biotech, Dorset, UK), glutamine (PAN-Biotech, Dorset, UK), HEPES buffer (PAN-Biotech, Dorset, UK), sodium heparin, and bovine retina-derived growth factor. The next day the medium was replaced to remove red blood cells.

### 2.9. Cell Treatment

Immediately before the cell treatment a freeze-dried aliquot of CE was dissolved in cell culture medium (M199, 5% FBS only) and then diluted to obtain different polyphenols concentrations expressed as μg GAE/mL culture medium. Empty NPs or CE-loaded NPs were freshly prepared in steryle phosphate buffered saline (PBS) and then diluted in culture medium to the concentrations mentioned in Section 3.5. P3–P4 adherent HUVECs in 96-well plates were incubated for 2 h or 24 h with CE diluted to the polyphenols concentrations of 2, 5 or 10 μg/mL GAE. In addition, HUVECs were incubated for 2 h or 24 h with the two types of empty NPs (from 0.0125 to 0.5 mg/mL of Ch derivative polymers) or CE-loaded NPs (CE concentration, 2 µg/mL GAE). Then, cells were washed with PBS and treated with 50 μM of commercial H_2_O_2_ for 1 h to induce oxidative stress. Cells in medium only were used as positive control. At the end of each treatment, cells were analyzed for viability and ROS production.

### 2.10. Cell Viability

After the treatment of HUVECs with the samples under testing or with H_2_O_2_, cell viability was evaluated by the WST-1 assay, based on the cleavage of tetrazolium salt (WST-1, 4-[3-(4-iodophenyl)-2-(4nitrophenyl)-2H-5-tetrazolium]-1,3-benzene disulfonate, Roche Applied Science, Mannheim, Germany) by mitochondrial dehydrogenases, present in viable cells. Briefly, at the end of the treatments, HUVECs were incubated with tetrazolium salt (10 μL/well) for 3 h at 37 °C, 5% CO_2_. Then, the formazan dye formed was quantified by measuring the optical density at 450 nm (reference wavelength 650 nm), with a multiplate reader (Thermo Scientific Multiskan FC Microplate Photometer, Waltham, MA, USA). The absorbance was directly correlated to the number of metabolically active cells and viability was expressed as percent of viable cells.

### 2.11. ROS Production

Intracellular ROS production was evaluated by ROS fluorescent probe 5-(and-6)-chloromethyl-2′,7′-dichloro-di-hydro-fluorescein diacetate, acetyl ester (CM-H2DCFDA) (Invitrogen, Carlsbad, CA, USA), a cell-permeable indicator for these compounds. Briefly, during the last 30 min of treatment with the samples under test or with H_2_O_2_, HUVECs were co-incubated with CM-H2DCFDA (10 μM/well) dissolved in PBS, in the dark at room temperature. ROS production was detected by measuring the increase in fluorescence over time by microplate reader (Thermo Scientific Fluoroskan Ascent Microplate Fluorometer, Waltham, MA, USA). Fluorescence was measured by excitation at 488 nm and emission at 510 nm.

### 2.12. Statistical Analysis

All data are presented as means ± SD (standard deviation). Three-six independent replicates of each experiment were carried out (3 ≤ *n* ≤ 6). The statistical significance of differences between two means was assessed by the Student’s t-test. Differences were considered significant, i.e., the null hypothesis was rejected, for *p* values lower than 0.05.

## 3. Results and Discussion

### 3.1. CE Characterization

Other authors reported that different species and varieties [30,31,32,33] of sweet cherries have high and variable contents of antioxidants and total polyphenols (TPC). In this work the autochthonous Tuscan variety of *Prunus avium* L., named Crognola, has been investigated for content in total antioxidant molecules by FRAP (Ferric Reducing Antioxidant Power) and Folin–Ciocalteu methods. When compared to TPC values from other cherry varieties of *Prunus avium* L. from southern Italy, the TPC of CE obtained from fresh fruits (402.5 ± 8.4 mg GAE/100 g fresh weight (FW)) turned out to be the uppermost [32,33]. After CE freeze-drying, the analysis with FRAP and Folin–Ciocalteu reagents always resulted in high antioxidant power (0.229 mg of Fe^2+^/mL) and TPC (26.7 μg/mL GAE per mg of dry weight), respectively. Recent in vitro studies have demonstrated the antioxidant and antitumor properties of *Prunus avium* L. derivatives [34,35,36,37,38]. The presence of high contents in polyphenols has made our CE interesting for studying its bioactive effect on HUVECs, before and after encapsulation into NPs.

### 3.2. NP Characterization

The physical characteristics of CE-loaded NPs based on quaternary ammonium chitosan (NP1) and S-protected thiolated derivative thereof (NP2), immediately after their preparation, are listed in Table 1. Neither the mean particle size nor the polydispersity index and the encapsulation efficiency (EE) appear to significantly depend on the type of either Ch derivatives from which the NPs were prepared. The ζ-potential values were positive in agreement with the presence of quaternary ammonium ions on the NP surface. The CE encapsulation efficiency was very high, demonstrating a high interactivity of the extracts with the polymers.

### 3.3. CE Stability Studies

**Figure 1:**
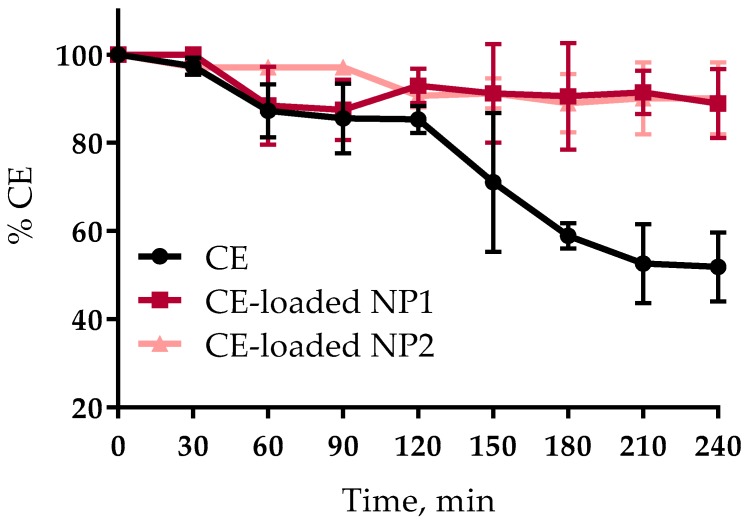
CE stability in simulated gastric fluids (SGFs).

The stability plots of CE, either free or encapsulated in NPs, in simulated gastric fluids (SGFs), are reported in Figure 1. Both NP types were able to protect the CE from degradation for at least 4 h, in fact, the encapsulated CE stability was around 90%, i.e., two-fold higher than that of the free CE. The results point out much more stability of CE encapsulated in either NP type in comparison with the plain CE. At the end of the stability study, NP1 and NP2 were checked for size. Both showed a slight size increase (NP1, 394.4 ± 8.8 nm; NP2, 379.9 ± 9.9 nm) not so marked as to exceed the upper size limit reported by Norris et al. [39] for the particles able to cross the intestinal barrier (500 nm). This data demonstrate that the CE encapsulation into NPs protects them from oxidation, thus preserving their therapeutic potential.

### 3.4. Ex Vivo Permeation Experiments

**Figure 2:**
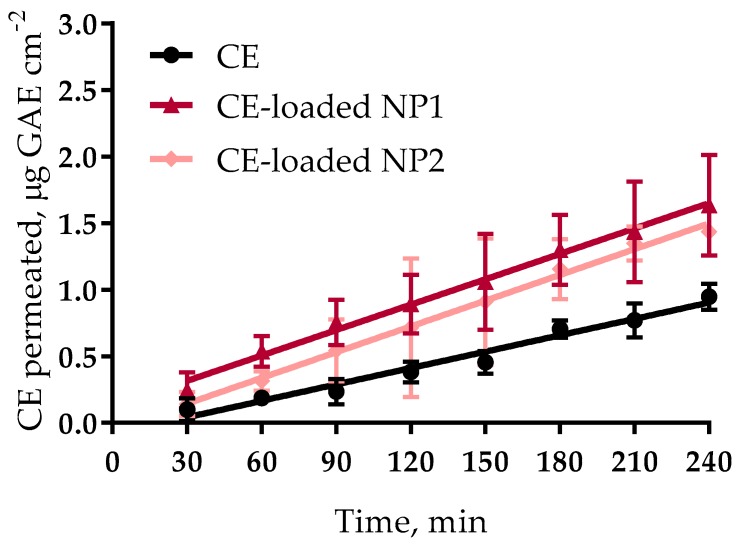
CE apparent permeation plots across excised rat intestine. CE = cherry extract; NP1 = NPs based on quaternary ammonium chitosan; NP2 = NPs based on quaternary ammonium S-protected thiolated chitosan; GAE = gallic acid equivalent.

For each permeation run, described in Section 2.7, a value of apparent permeability coefficient, P’_app_, for permeant across the excised rat intestinal mucosa was calculated from the following equation:

P’_app_ = dM/dt 1/(A C_0_) (2)

where dM/dt 1/A, the permeation flux, is the slope of the linear portion of the cumulative amount permeated per unit surface area vs time plot, and C_0_ is the CE concentration introduced into the donor phase, i.e., the CE concentration in the regenerated dispersion (C_0_ = 15.5 µg/mL GAE). The single P’_app_ values were averaged to calculate the mean apparent permeability P_app_ (*n* ≥ 3). The mean cumulative amount permeated per unit area in any given time was calculated to plot each permeation profile and determine T_4 h_, i.e., the cumulative transport per unit area over the whole time of experiment (4 h). For NPs that produced a significant P_app_ increase, this was measured by the enhanced permeability ratio (EPR), defined as the ratio between each of the P_app_ values obtained with the NPs and that obtained with the control plain CE solution in PB. The apparent permeation plots for CE, seen in Figure 2, are all significantly linear (*r*^2^ > 0.9), in agreement with our hypothesis for application of the above Equation (2). Such a linearity allowed the calculation of a P_app_ parameter for each formulation, useful for comparative purposes. The relevant data, listed in Table 2, show that both NP types had the same impact on the CE apparent permeation parameters; in fact, no significant differences were seen between the respective flux, P_app_, and T_4h_ values, all of which were significantly higher than the corresponding values for the control, i.e., the free CE. Since the concentration in the donor was the same in all cases (15.5 μg/mL GAE) the results point to much more aptitude of NPs to cross the excised intestinal wall compared to the free CE, with no significant difference between the two NP types. The results agree with those obtained previously, with the same NP types, regarding the promotion of the apparent permeability of a macromolecular drug (Fluorescein (FD4)) across the excised intestine [19]. Hence, cherry-loaded NPs show promise of both promoting the intestinal absorption of CE and protecting it from degradation in the stomach.

### 3.5. Effect of Empty NP, Free CE, and NP-Loaded CE on HUVEC Viability

**Figure 3:**
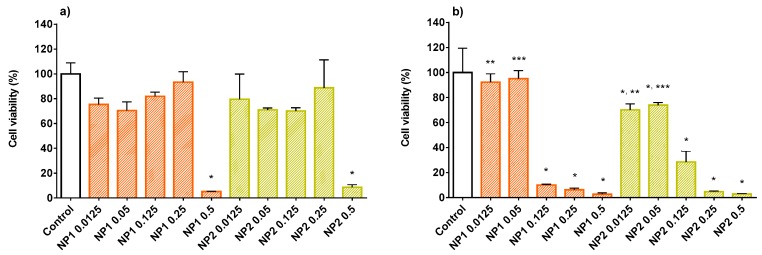
Human umbilical vein endothelial cell (HUVEC) viability after 2 h (**a**) or 24 h (**b**) of incubation with empty NP1 or NP2 (0.0125 to 0.5 mg/mL) in culture medium. Data are expressed as % viable cells compared to 100% of control (untreated cells). *, significantly different from control; **, ***, significantly different from each other.

**Figure 4:**
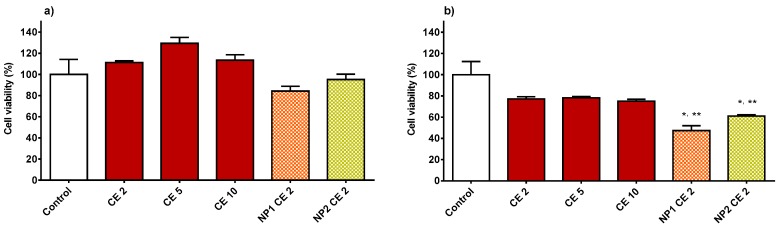
HUVEC viability after 2 h (**a**) or 24 h (**b**) of incubation with free CE (2, 5, 10 µg/mL GAE) and CE-loaded NP1 or NP2 (CE concentration, 2 µg/mL GAE) in culture medium. Data are expressed as % viable cells compared to 100% of control (untreated cells). *, significantly different from control; **, significantly different from each other.

Numerous studies have tested polyphenols derived from natural products in in vitro experiments with HUVECs, related to vascular dysfunction [20,21,22,40]. In the present work we have investigated in vitro the beneficial properties of CE, free or encapsulated in NPs. CE polyphenolic concentrations of 2, 5 and 10 μg GAE per mL of HUVEC culture medium were chosen for viability studies. Before testing CE, the cytotoxicity of empty NP1 and NP2 on HUVECs was studied. Figure 3 reports the viability results after a cell pre-treatment of 2 h (a) or 24 h (b) with empty NPs at different concentrations (0.0125, 0.05, 0.125, 0.25, and 0.5 mg/mL). After 2 h (Figure 3a) no cytotoxicity effect for both types of empty NPs was found, except for the concentration of 0.5 mg/mL, which significantly reduced the viability. No significant difference between the two types of NPs was found. On the other hand, after 24 h (Figure 3b) only at the lower concentrations (0.0125 and 0.05 mg/mL) the cell viability did not drop under 70%, and NP2 turned out to be more toxic to HUVECs (viability, 72.0 ± 3.9%) than NP1 (viability, 93.7 ± 6.1%). Therefore, the maximum non-toxic concentration of 0.05 mg/mL was chosen for studying the viability of HUVECs exposed to NP1 or NP2-loaded CE, in which the concentration of encapsulated CE polyphenols was 2 μg/mL GAE. In Figure 4 the results for the viability of cells exposed to free CE are shown. As can be seen, after either 2 h (4a) or 24 h (4b) of pre-treatment, no toxic effect was found at whichever of the concentrations tested. On the other hand, NP1- and NP2-loaded CE maintained its non-cytotoxicity after 2 h but not after 24 h, when the cell viability was significantly reduced (down to 47.3 ± 8.0%). A significant difference between the two types of NP-loaded CE is seen in Figure 4b, corresponding to 24 h of incubation, where CE-loaded NP2 appears to be significantly less cytotoxic than CE-loaded NP1. For these reasons altogether, in the subsequent experiments no cell treatment was protracted for more than 2 h and the NP concentration of 0.05 mg/mL, corresponding to 2 μg/mL GAE was used. Indeed, at the lowest concentration tested, i.e., 0.0125 mg/mL, the CE concentration encapsulated, i.e., 0.5 μg/mL GAE, would be too low to evidence any therapeutic effect.

### 3.6. Free CE and NP-Loaded CE Protective Effect from Oxidative Stress

**Figure 5:**
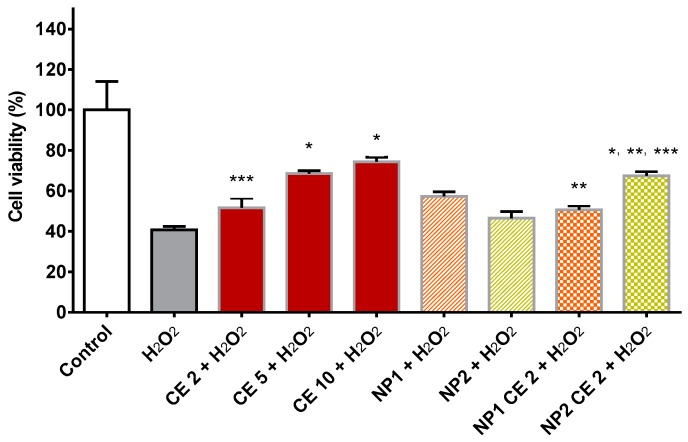
HUVEC viability after 2 h of pre-treatment with free CE (2, 5, 10 µg/mL GAE), empty NP1 or NP2, or CE-loaded NP1 or NP2 (CE concentration 2 µg/mL GAE), and the subsequent treatment with 50 μM H_2_O_2_ for 1 h. Data are expressed as % viable cells compared to negative control (H_2_O_2_). *, significantly different from H_2_O_2_; two means at comparison are indicated by the same mark (** or ***) if their difference is significant.

Vascular oxidative stress contributes to the mechanisms of vascular dysfunction and is implied in a number of cardiovascular pathologies [41]. A number of in vitro studies have been reported on the antioxidant properties of *Prunus avium* L., which is known to prevent chronic diseases [36,37,38,42]. The presence of polyphenolic molecules made CE interesting as means to protect cells from oxidative stress. Previously, Serra et al. [36] showed an antiproliferative activity of human cancer cells following pre-incubation with different varieties of Portuguese sweet cherries. Matias et al. [37] demonstrated that a 2 h pre-treatment with cherry extracts was effective in alleviating the oxidative stress caused by H_2_O_2_-induced injury in neuronal cells. In this study we have evaluated the CE influence on HUVEC viability after H_2_O_2_-induced oxidative stress. In Figure 5 data on HUVEC viability after 2 h pre-treatment with free or NP-loaded CE and subsequent treatment with H_2_O_2_ are reported. Data show that HUVECs treatment with H_2_O_2_ significantly reduced viable cell number compared to control (cells with medium). Data also show that a pre-incubation with free CE for 2 h at the concentrations of 5 and 10 μg/mL GAE significantly reduced the H_2_O_2_-induced drop in viable cell number. These results demonstrated the ability of free CE to protect cells from oxidative stress in a small range of polyphenolic concentrations. As regards empty NP, they seem to have enhanced cell viability after oxidative treatment, even though this effect was not statistically significant, due to data variability. Figure 5 also shows that CE-loaded NP2 (2 μg/mL GAE) significantly protected HUVECs from oxidative stress, while the same concentration of free CE had no effect. The data suggest that the higher ability of CE-loaded NP2 to act as an antioxidant is probably due to a synergistic effect between CE and NP2. Since no protective effect was observed with NP1-encapsulated CE, we thought that the antioxidant effect was enhanced by the presence, on the NP2 surface, of protected thiol groups, which are known to act as strong reducing groups [43].

### 3.7. Antioxidant Activity of Free CE or CE Loaded in NPs as Evaluated from ROS Production

**Figure 6:**
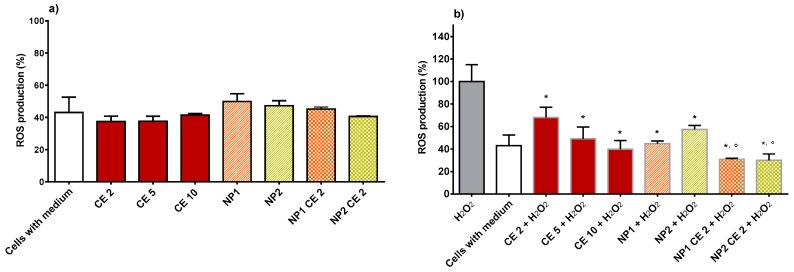
(**a**) shows reactive oxygen species (ROS) production in HUVECs treated with free CE (2, 5, 10 µg/mL GAE), empty NP1 or NP2, or CE-loaded NP1 or NP2 (CE concentration 2 µg/mL GAE) for 2 h. (**b**) represents ROS production after 2 h of pre-treatment with free CE (2, 5, 10 µg/mL GAE), empty NP1 or NP2, or CE-loaded NP1 or NP2 (CE concentration 2 µg/mL GAE) and subsequent treatment with 50 μM H_2_O_2_ for 1 h. Data are expressed as % ROS production compared to 100% (cell treated with H_2_O_2_). * Significantly different from H_2_O_2_*; °* significantly different from CE 2 + H_2_O_2_.

Figure 6a represents basal ROS production of cells treated with CE, either free or loaded in NP, compared with control (cells with medium). No significant difference between the different treatments is observed. Figure 6b shows data relative to ROS production by HUVECs treated with the suspensions under study and, subsequently, with H_2_O_2_. As can be seen, H_2_O_2_ significantly increased intracellular ROS production in HUVECs. After 2 h of pre-treatment with CE at the concentrations of 2, 5, and 10 μg GAE/mL, a significant % reduction of H_2_O_2_-produced ROS is evidenced. Pre-treatment with empty NP1 or NP2 significantly reduced ROS production after oxidative stress, nevertheless, CE-loaded NP1 or NP2 were more effective than the respective empty NPs. Moreover, both empty and loaded NPs had more antioxidant effects than the free CE at the same concentration as that contained in NPs, i.e., 2 μg/mL GAE. These results suggest that CE polyphenols are able to inhibit ROS production, and more so when encapsulated in NPs. This is probably due to the ability of NPs to protect CE from degradation, as shown in Figure 1. The data shown in Figure 6b for CE-loaded NP1 and NP2 apparently disagree with the data in Figure 5, where only CE-loaded NP2 shows the ability to increase the viability of H_2_O_2_ stressed HUVECs. However, it should be considered that the probe used to quantify ROS production has a higher affinity for viable cells [44]. As Figure 5 shows, a higher viable cell fraction is found in HUVECs pre-treated with CE-loaded NP2 compared with those pre-treated with CE-loaded NP1. Then, assuming even effectiveness, the observation of a lower ROS concentration is expected with HUVECs pre-treated with CE-loaded NP1. In fact, a not significant difference in ROS production is observed, in Figure 6b, between HUVECs cells pretreated with CE-loaded NP1 or NP2. From the above reasoning, the data in Figure 5 are confirmed, i.e., it is concluded that CE-loaded NP2 has greater antioxidant ability than CE-loaded NP1, probably thanks to a synergistic effect of CE and the protected thiols present on the NP2 surface. No such effect was seen in a previous study by our group [18], where red grape seed extract, encapsulated in NPs based on Ch derivatives different from the present ones, was tested on endothelial progenitor cells. Probably, the lack of antioxidant effect of the previous NPs should be ascribed to the thiols present on the NP surface not being protected. It is known indeed that the non-protected thiol groups are instable and prone to degradation in the GI [45].

## 4. Conclusions

In the present study, the goal of increasing the ability of cherry extract from *Prunus avium* L. growing in Tuscany to protect endothelial cells from oxidative stress, thanks to its encapsulation in nanoparticles based on chitosan derivatives, was obtained. The two nanoparticle types studied were only different for the presence or absence of superficial protected thiol groups. Therefore, the differences in biological properties between the two nanoparticle types were ascribed to the chemical surface differences. Both types have shown an ability to protect the antioxidants contained in the extract from the degradation they undergo in the GI tract. However, the nanoparticles containing protected thiol groups have shown a higher effectiveness in protecting the endothelial cells from oxidative stress. Considering that the fresh cherry fruit is only available a few days a year, it is understood that the nanoparticles based on protected thiolated chitosan, in addition to improving the effectiveness of the encapsulated extract, also allow the possible beneficial effects from cherry consumption not to be limited by the seasonality of the fruit.

## Figures and Tables

**Table 1:** Characteristics of nanoparticle (NP)-loaded cherry extract (CE).

NP Type	Nanoparticle Size, nm (Polydispersity Index)	ζ, mV	EE, %
CE-loaded NP1	344.9 ± 17.8 (0.52 ± 0.08)	14.8 ± 0.3	78.4 ± 4.5
CE-loaded NP2	339.9 ± 68.2 (0.50 ± 0.09)	15.8 ± 0.5	79.8 ± 0.6

NP1 = NPs based on quaternary ammonium chitosan; NP2 = NPs based on quaternary ammonium S-protected thiolated chitosan; EE = encapsulation efficiency.

**Table 2:** Data on CE permeation across excised rat jejunal epithelium from phosphate buffer (PB, PH = 7.4) containing CE alone or encapsulated in NPs (15.5 µg/mL gallic acid equivalent (GAE).

**Formulation**	**Flux 10^3^ (μg·cm^−2^·min^−1^)**	**P_app_ 10^4 a^ (cm·min^−1^)**	**EPR ^b^**	**T_4 h_^c^ (μg·cm^−2^)**
CE	4.10 ± 0.28	2.64 ± 0.02	-	0.95 ± 0.10
CE-loaded NP1	6.36 ± 0.23 *	4.10 ± 0.15 *	1.55	1.63 ± 0.38 *
CE-loaded NP2	6.45 ± 0.20 *	4.16 ± 0.13 *	1.58	1.43 ± 0.06 *

^a^ Apparent permeability. ^b^ Enhanced permeability ratio (ratio of P_app_ to the value for the control, CE). ^c^ Cumulative transport over the whole experimental time (4 h). * Significantly different from control, CE.
